# Assessment of Bone Mineral Density in Type 2 Diabetes: A Cone Beam Computed Tomography (CBCT) Study

**DOI:** 10.7759/cureus.28035

**Published:** 2022-08-15

**Authors:** Chinmayee Dahihandekar, Sweta G Pisulkar, Seema Sathe, Surekha Godbole, Akansha V Bansod, Hetal Purohit

**Affiliations:** 1 Prosthodontics, Sharad Pawar Dental College, Wardha, IND

**Keywords:** bone mineral density, implant therapy, edentulous mandible, cone beam computed tomography, type 2 diabetes mellitus

## Abstract

Introduction

The main purpose of the study was to assess and compare bone mineral density (BMD) at prospective implant sites in the mandible in type 2 diabetes mellitus (T2DM) and non-diabetic patients using cone beam computed tomography (CBCT).

Material and methodology

A total of 40 patients were included in this type of cross-sectional study. They were divided into two groups, A and B, according to their haemoglobin A1c values. Group A consisted of patients with HbA1c between the range of 6.1%-8% and group B had patients with no history of T2DM. CBCT scans were made of the mandibular arches of both the patients to evaluate the BMD at lingual and buccal cortical plates and the trabecular regions in two successive slices with the assistance of PlanMeca Romexis software (PlanMeca Romexis®, Helsinki, Finland). The Shapiro-Wilk test was used to determine the normality of continuous data. The Mann-Whitney U test was used to compare the groups.

Results

There were no differences that were statistically significant between the two cohorts according to the Mann-Whitney U test at buccal cortical plate points 1 and 2. However, the diabetes group's mean bone density at implant sites-A, B, C, D, and E at trabeculae points 1 and 2 was considerably (7p>0.001) lower than the non-diabetic groups. The mean bone density of the diabetes group was marginally but significantly (p=0.009) lower than the non-diabetic group at lingual cortical plates.

Conclusion

Individuals with type 2 diabetes mellitus show significantly lower bone mineral density in the lingual cortical plate and trabecular region, however, implant therapy can be performed with certain mentioned guidelines in such regions. In the buccal cortical region, the bone mineral density is seen to be unaffected.

## Introduction

India, being a developing country with a young population, has a significant share of the geriatric population. Diabetes mellitus, dubbed the "silent killer" [1], reveals the harsh reality of our country. According to the "2016 Ministry of Statistics and Programme Implementation (MOSPI) Report," India's total senior population (those over 60 years old) is 103.9 million, accounting for 8.6% of the total population. The possibility of restoring edentulous mouth surfaces in clinics is increasing as people live longer. In prosthodontics, endosseous implants are the greatest therapy option for removable denture prostheses for partially edentulous and completely edentulous patients, and they are proven successful [2]. However, some systemic disorders, such as diabetes mellitus, which causes poor healing and has a recognised effect on bone, might also have a detrimental impact on this success rate [3].

Osseointegration is affected by diabetes mellitus, which directly affects the overall survival rate and prognosis of implant therapy. Diabetes-related changes in micro-vascularization result in a lowered response of immunity and a decrease in the remodelling of bone mechanisms. Because of the persistent inflammation, hyperglycaemia in diabetics inhibits osteoblastic activity and changes parathyroid hormone responses, which show the reduced formation of collagen during the formation of callus. Apoptosis is initiated in bone-lining cells and increases osteoclastic activity [4].

Type 2 diabetes mellitus (T2DM) is linked with insulin's inability to respond to a glucose increase. Type 1 diabetes mellitus (T1DM) is linked with a decrease in the production of β cells in the pancreas islet of Langerhans. In her study, Dr. Mishaela R. Rubin claimed that in type 2 diabetes, the pattern of the bone in the trabeculae is altered by the deterioration of the bone in the trabecular region, which promotes bone fractures [5].

The definition of bone density is mentioned in literature as the quantity of mineral matter per square centimetre of bone and is considered the most crucial indicator of dental implant success. Bone mass density is reduced by T1DM, but there is no conclusion on evidence of how T2DM affects bone density [6]. Certain researchers have found that bone mineral density affects implant insertion success and prognosis [7].

To guarantee implant placement success, the pre-surgical assessment should be thorough and precise in terms of bone density and width, as well as knowing the neighbouring anatomy of the surrounding area, in addition to examining the patient's medical history [8]. Cone beam computed tomography is included amongst them since it provides advanced imaging as well as a complete comprehension of the underlying structures while also overcoming some of the disadvantages of other traditional modalities [9].

The study served the purpose of evaluation of BMD of the prospective implant sites in the edentulous mandible in patients with T2DM and compared them with non-diabetic patients to estimate the success and prognosis of the implant placement procedure.

## Materials and methods

This cross-sectional study was conducted at Sharad Pawar Dental College and Hospital in Sawangi, in collaboration with the Departments of Prosthodontics and Oral Diagnosis, Medicine, and Radiology. The study's permission was acquired from the Ethical Committee (DMIMS) Ref No. DMIMS (DU)/IEC/Aug-19/8276. A total of 40 completely edentulous patients from the outpatient department were selected according to the sample size, which was calculated for the mentioned research, and were bifurcated into two groups calculated by the method of difference of means. The duration of the study was two years from October 2019 to October 2021. Sample size calculation formula: N=2 s2 (Zß + Zα/2)2/D2. The first group consisted of 20 patients who were diagnosed with T2DM at least two weeks ago. They had the inclusion criteria that their age was within the range of 50-65 years, they had HbA1c in the range of 6.1%-8% and they consented to participate in the study. The second group consisted of the control group with an age range of 50-65 years and no history of diabetes mellitus. The study and the procedures involved were explained to the patients in a language they understood before the beginning of the procedure. The 40 patients were segregated into two groups, namely, group A and group B depending upon the history that was recorded. To confirm the diagnosis of diabetes and to determine glycaemic control, an HbA1c analysis of all subjects was done. The data were collated and classified into the following categories [5,8] (Table 1).

**Table 1:** Glycated haemoglobin values categorised under control values.

HbA1c levels	Glycaemic control
≤6.5%	Non-diabetics
<8.0%	Well-controlled diabetics
<10%	Moderately controlled
≥10.1%	Uncontrolled

**Figure 1:**
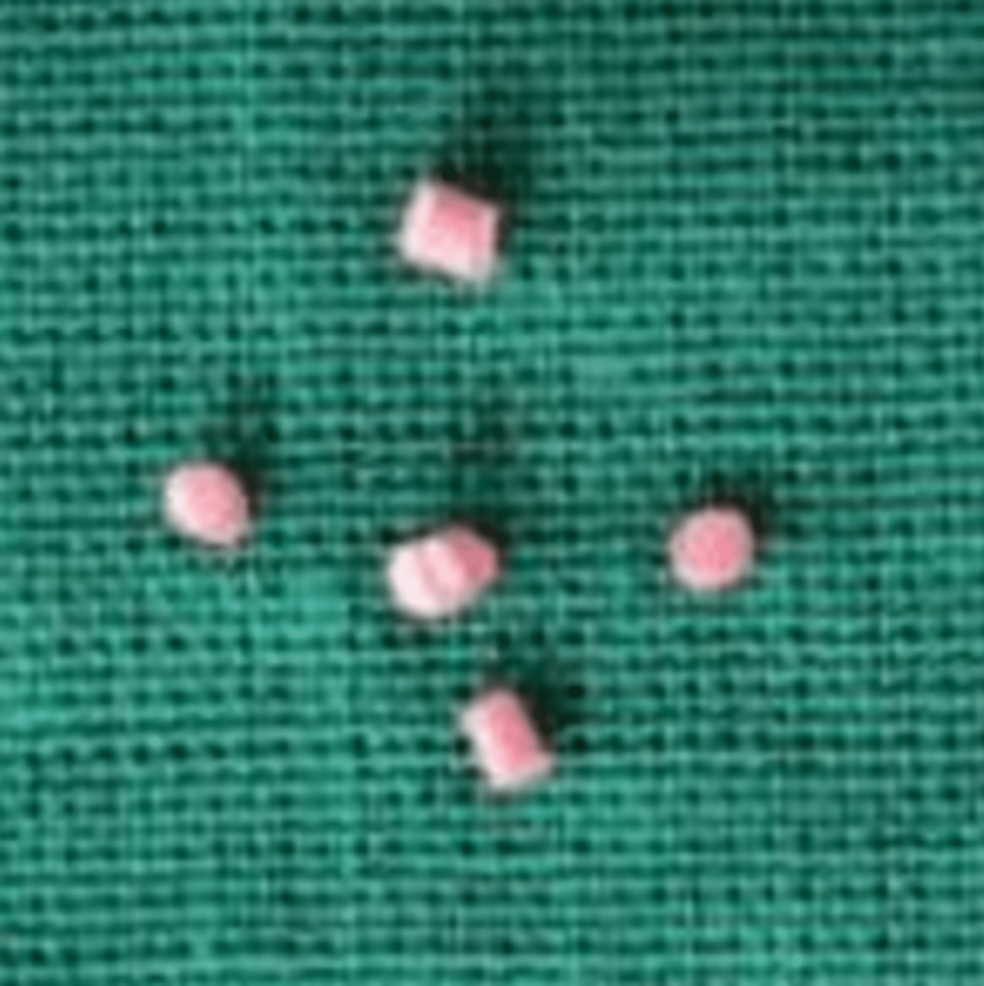
Gutta-percha cones (1 × 1 mm in dimension).

**Figure 2:**
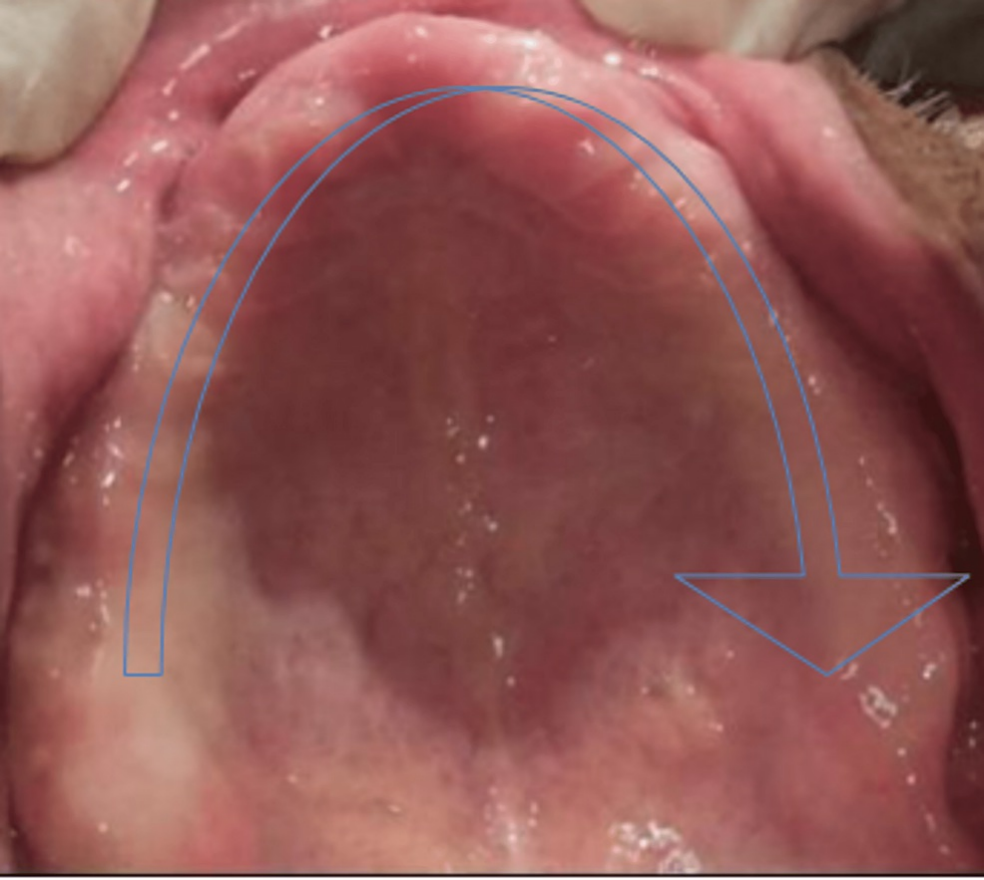
Controlled diabetic type 2 patient with a completely edentulous maxillary arch whose glycaemic haemoglobin was checked and found to be in the range of 6.1%–8% devoid of any pathology and tooth remnants. Completely edentulous maxillary arch without any deformity.

**Figure 3:**
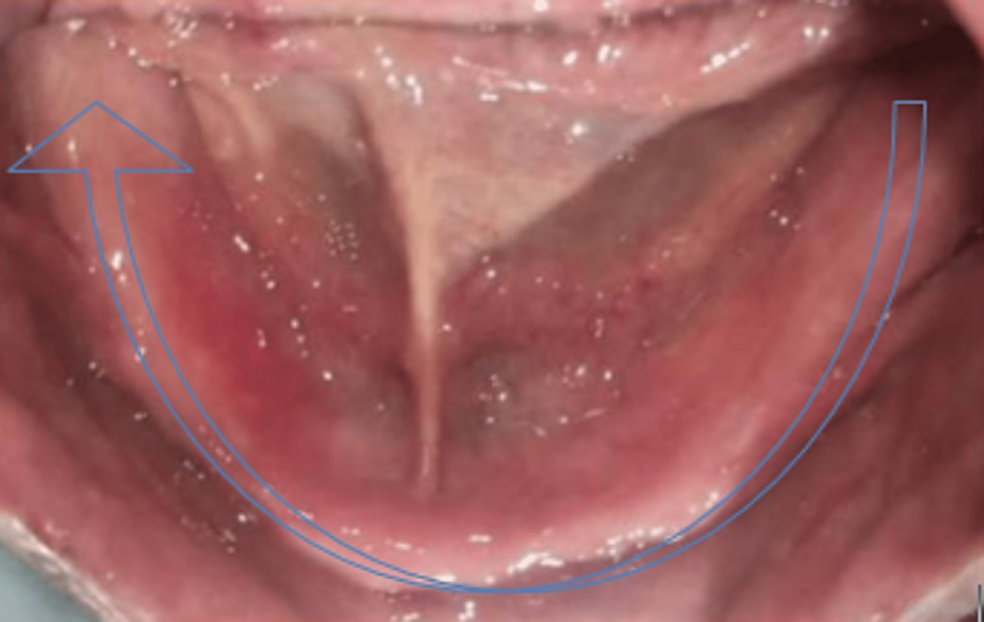
Controlled diabetic type 2 patient with a completely edentulous mandibular arch whose glycaemic haemoglobin was checked and found to be in the range of 6.1%–8% devoid of any pathology and tooth remnants. Completely edentulous mandibular arch without any deformity.

**Figure 4:**
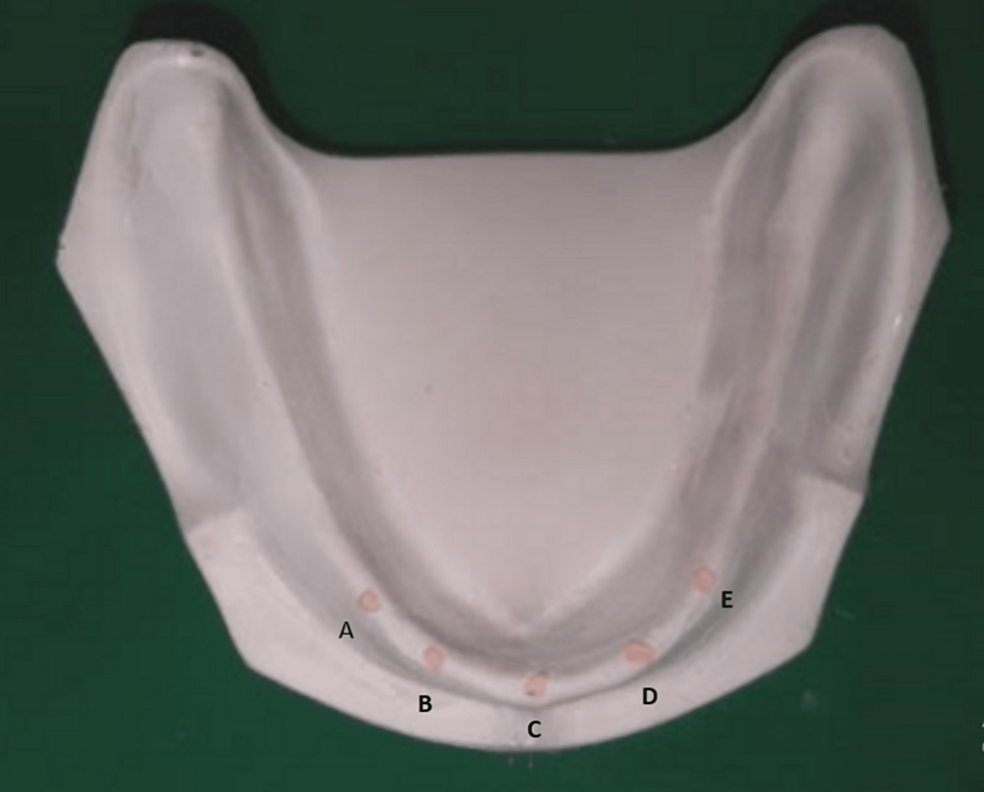
A, B, C, D, and E implant positions, according to Misch, are marked on the diagnostic cast, and a stent is fabricated. According to Misch, five positions for implant placement have been given to improve the success rate of the procedure. These positions are designated as A, B, C, D, and E as given in the figure.

**Figure 5:**
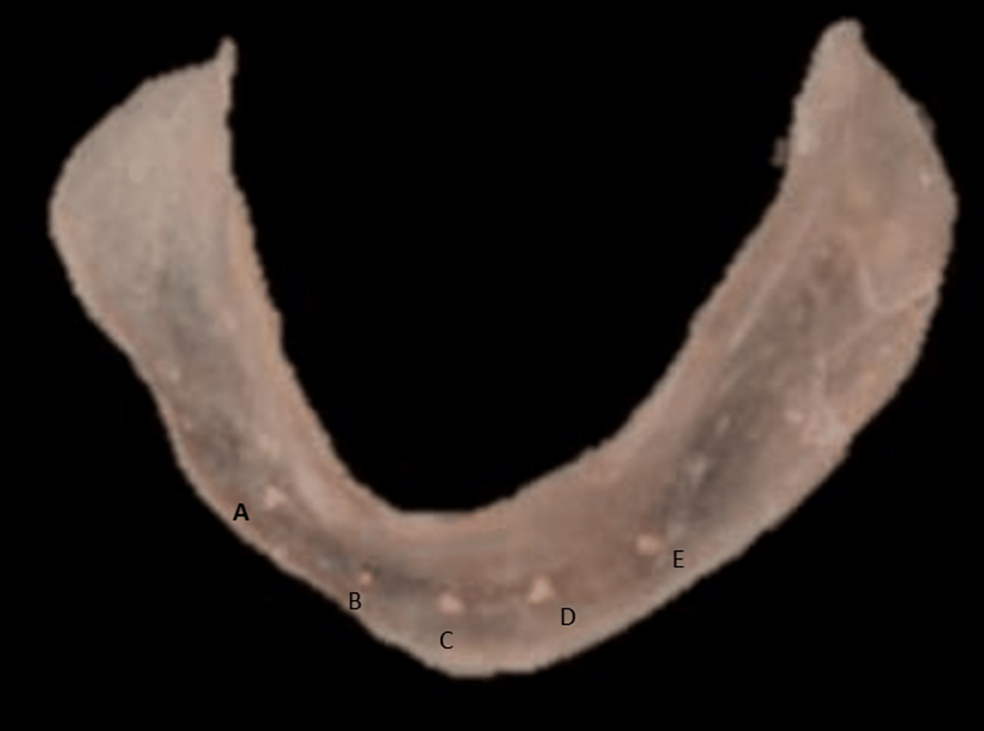
A, B, C, D, and E implant positions, according to Misch, are marked on the diagnostic cast and gutta-percha cones are fixed. The stent was prepared by incorporating the gutta-percha cones placed earlier in the cast as positions described by Misch as A, B, C, D, and E.

For each of the 40 patients, an impression for diagnosis was taken and casts were poured. The distance between the mental foramina was measured and divided into five equal columns. Implant sites on the diagnostic castings were designated at five sites of prospective implant placement at locations A-B-C-D-E according to prospective implant sites as advised by Misch (2008) [8]. The 1 × 1 mm of gutta-percha cones were cyanoacrylate-fixed in the cast and then inserted into the autopolymerizing resin (Figures 1, 2, 3, 4, 5).

**Figure 6:**
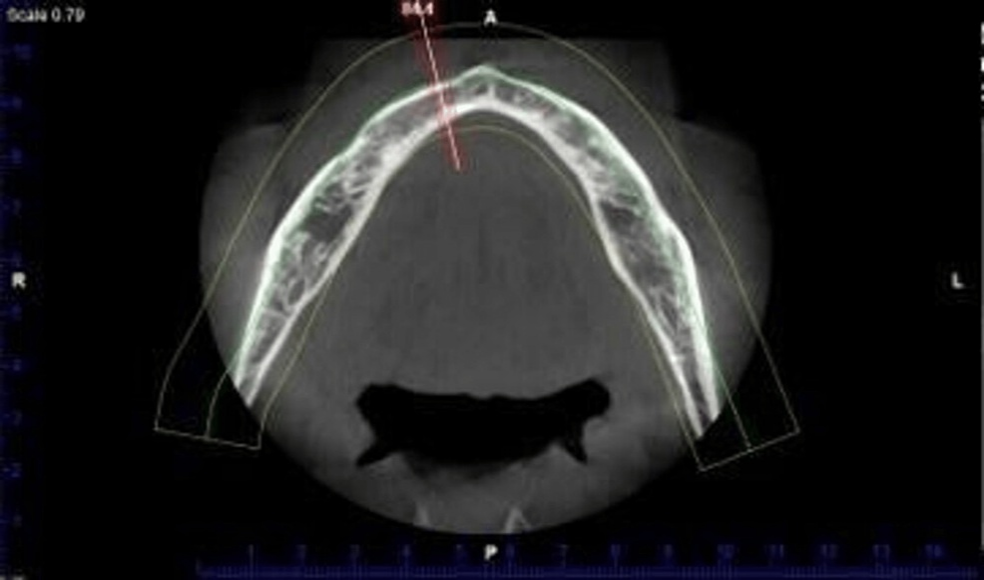
CBCT scans of the patients with PlanMeca Romexis software under ideal conditions of 84 kV and 14 mA. The stent was placed on the edentulous mandible and a CBCT scan was made. CBCT: cone beam computed tomography.

**Figure 7:**
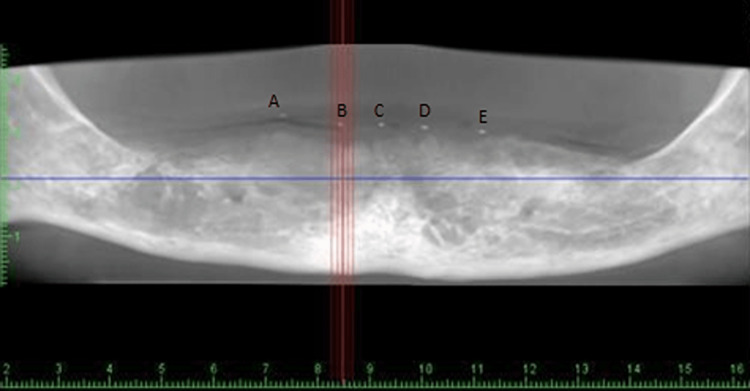
CBCT scan of the edentulous mandible after placing the stent with embedded gutta-percha cones at prospective implant sites. With the assistance of radio-opaque gutta-percha cones, one can clearly visualise the area of the Misch prospective implant sites A, B, C, D, and E on the CBCT scan. CBCT: cone beam computed tomography.

**Figure 8:**
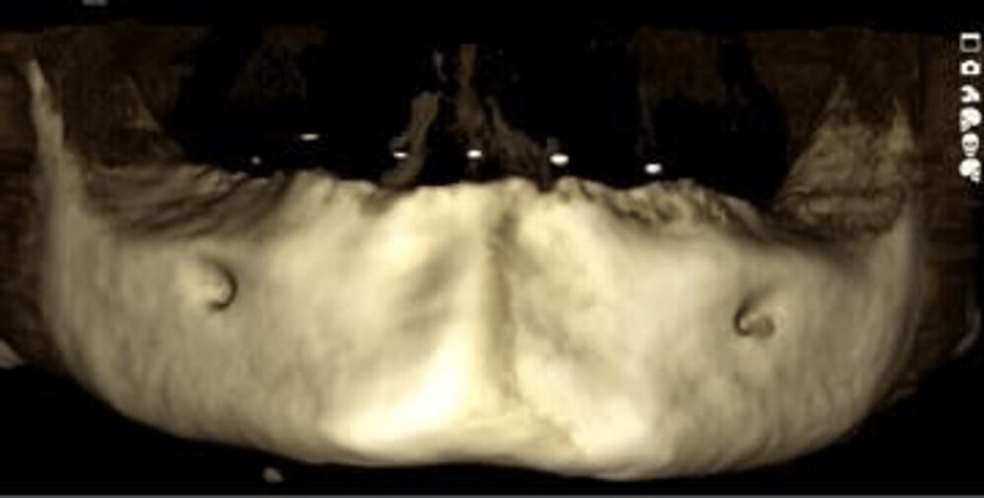
CBCT scan and 3D imaging of the edentulous mandible after placing the stent with embedded gutta-percha cones at prospective implant sites. The gutta-percha cones can be visualised on the edentulous mandible as shown in the figure. CBCT: cone beam computed tomography.

When the stent with included gutta-percha cones was inserted in the mandibular arch of the patient, bone density was assessed using CBCT with controlled T2DM patients and non-diabetic patients with the aid of the PlanMeca Romexis Promax machine (PlanMeca Romexis®, Helsinki, Finland). PlanMeca Romexis software (PlanMeca Romexis®, Helsinki, Finland) was utilised in this project (Figures 6, 7, 8).

**Figure 9:**
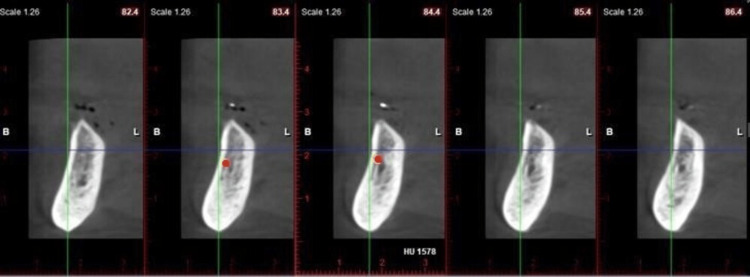
Evaluating the bone mineral density in the Hounsfield unit after placing the cursor at the lingual cortical plate at two successive sections, for example, 1578 HU. The cursor can be placed with the help of the red dot, which can be seen in the figure. The cursor, when placed, records the Hounsfield unit (HU) of the respective site.

In the areas of the buccal cortical plate, lingual cortical plate, and trabeculae in the mandibular arch in the sites of prospective implant placement, densities were assessed in two successive sections by placing a cursor, and their mean was derived (Figure 9).

Later, this means was utilised to compare the density of non-diabetic patients' edentulous mandibular arches at potential implant sites to that of managed type 2 diabetes patients' edentulous mandibular arches.

For all 40 patients, identical settings of 14 mA and 84 kV were employed in the CBCT equipment. The Shapiro-Wilk test was used to determine the normality of continuous data. Non-parametric tests were performed to analyse the data since it didn’t follow a normal distribution. The Mann-Whitney U test is a non-parametric test that compares two groups without assuming that their values are distributed regularly. A p<0.05 value was deemed significant statistically.

## Results

A total of 40 participants were included in the study throughout all the phases of the study. The mean, median, and standard deviation were used to represent the values. The non-parametric tests were performed to analyse the data since it didn’t follow a normal distribution. The Mann-Whitney U test is a non-parametric test that compares two groups without assuming that their values are distributed regularly. A p<0.05 value is deemed significant statistically. The bone mineral density at implant sites, A, B, C, D, and E (Figure 4) between the two groups of diabetic patients were compared (Table 2) and non-diabetic patients were also tabulated (Table 3). 

**Table 2:** Comparison of the bone mineral density at implant sites, A, B, C, D, and E between the two groups of diabetic patients. For no bias in the recording of the Hounsfield unit (HU) at prospective implant sites at A, B, C, D, and E, two sites were selected on either side of one and two on two successive sections of CBCT. Later on, an average was calculated to avoid error or manipulation of results. This table has been made for patients with diabetes mellitus. CBCT: cone beam computed tomography, T1: trabecular section 1, T2: trabecular section 2, BC1: buccal cortical section 1, BC2: buccal cortical section 2, LC1: lingual cortical section 1, LC2: lingual cortical section 2.

Variables	Site	N	Mean	SD	Median	P value
A	T1	20	446.39	8.45	444.58	<0.001
	T2		446.99	8.89	444.23	<0.001
	BC1		1264.57	6.00	1265.65	0.183
	BC2		1264.57	5.95	1266.13	0.221
	LC1		1479.08	44.86	1458.73	0.009
	LC2		1479.13	44.70	1458.90	0.007
B	T1	20	445.75	8.29	443.95	<0.001
	T2		447.30	8.94	443.29	<0.001
	BC1		1264.48	5.11	1265.65	0.102
	BC2		1264.50	5.18	1266.13	0.114
	LC1		1481.68	11.18	1480.59	0.009
	LC2		1481.69	11.15	1480.67	0.007
C	T1	20	445.79	8.87	442.01	<0.001
	T2		446.38	9.3	442.09	<0.001
	BC2		1266.89	4.13	1268.56	0.968
	LC1		1481.33	11.47	1480.59	0.008
	LC2		1481.38	11.41	1480.65	0.007
D	T1	20	445.09	8.36	442.01	<0.001
	T2		446.21	9.11	442.09	<0.001
	BC1		1266.41	5.45	1267.76	0.602
	BC2		1266.59	5.51	1267.06	0.640
	LC1		1481.68	11.18	1480.59	0.009
	LC2		1481.69	11.15	1480.67	0.007
E	T1	20	445.64	8.17	443.04	<0.001
	T2		446.22	8.62	442.85	<0.001
	BC1		1266.06	6.47	1265.65	0.478
	BC2		1266.22	6.43	1266.13	0.485
	LC1		1481.68	11.18	1480.59	0.009
	LC2		1481.69	11.15	1480.67	0.007

**Table 3:** Comparison of the bone mineral density at implant sites, A, B, C, D, and E between the two groups of non-diabetic patients. For no bias in the recording of the Hounsfield unit (HU) at prospective implant sites at A, B, C, D, and E, two sites were selected on either side of one and two on two successive sections of CBCT. Later on, an average was calculated to avoid error or manipulation of results. This table has been made for patients with diabetes mellitus. CBCT: cone beam computed tomography, T1: trabecular section 1, T2: trabecular section 2, BC1: buccal cortical section 1, BC2: buccal cortical section 2, LC1: lingual cortical section 1, LC2: lingual cortical section 2.

Variables	Site	N	Mean	SD	Median	P value
A	T1	20	493.74	7.31	495.93	<0.001
	T2		493.94	7.32	495.47	<0.001
	BC1		1267.11	4.05	1266.71	0.183
	BC2		1267.28	3.86	1267.44	0.221
	LC1		1481.68	11.18	1480.59	0.009
	LC2		1484.69	11.17	1499.52	0.007
B	T1	20	490.92	8.73	489.94	<0.001
	T2		490.97	8.85	489.82	<0.001
	BC1		1267.76	3.98	1257.18	0.102
	BC2		1267.59	3.81	1267.82	0.114
	LC1		1479.08	44.86	1458.73	0.009
	LC2		1379.13	44.70	1458.90	0.007
C	T1	20	490.01	8.20	489.76	<0.001
	T2		490.01	8.23	498.90	<0.001
	BC1		1267.11	4.05	1266.71	0.841
	BC2		1267.28	3.86	1267.44	0.968
	LC1		1478.56	44.79	1458.27	0.008
	LC2		1478.77	44.66	1458.65	0.007
D	T1	20	490.77	7.57	490.49	<0.001
	T2		490.91	7.43	490.29	<0.001
	BC1		1267.46	3.98	1267.18	0.602
	BC2		1267.59	3.81	12676.92	0.640
	LC1		1479.08	44.86	1458.73	0.009
	LC2		1479.13	44.70	1458.90	0.007
E	T1	20	490.71	6.7	489.7	<0.001
	T2		490.99	6.8	489.10	<0.001
	BC1		1267.75	4.4	1267.18	0.478
	BC2		1267.93	4.16	1267.92	0.485
	LC1		1479.08	44.86	1458.73	0.009
	LC2		1479.03	44.70	1458.90	0.007

The P-value derived from the Mann-Whitney U test; significant at p<0.05. In the present study, there were no differences that were statistically significant between the two cohorts according to the Mann-Whitney U test at buccal cortical plate points 1 and 2. However, the diabetes group's mean bone density at implant site-A at trabeculae point 1 (446.39 ± 8.45) was considerably (p>0.001) lower than the non-diabetic group's (493.74 ± 7.31). At trabeculae point 2, the diabetes group (446.99 ± 8.89) had substantially lower bone density (p>0.001) than the non-diabetic group (493.94 ± 7.32). The mean bone density of the diabetes group (1479.08 ± 44.86) was marginally but substantially (p=0.009) lower than the non-diabetic group (1481.68 ± 11.18) at lingual cortical plate point 1. At lingual cortical plate point 2, the mean bone density of the diabetic group (1479.13 ± 44.70) was slightly but significantly (p=0.007) lower than the non-diabetic group (1481.69 ± 11.15). Similar results were observed with the rest of the sites as mentioned in the tabular data (Table 2, 3).

## Discussion

India, being a developing nation has its fair contribution to the elderly population [10]. The older age group will gain the most if implant prosthodontics becomes a more commonly acknowledged therapeutic choice for the replacement of missing teeth. One of the most critical aspects that might impact the prognosis and integration of implants is proper patient selection for dental implant therapy [11].

Diabetes is expected to impact 72.96 million people in India's population [12]. In urban areas, the frequency ranges from 10.9% to 14.2%, but in rural India, the prevalence ranged from 3.0% to 7.8% among those aged 20 and over, with a much greater proportion among the elderly over 50.64 [13]. Increased production of cytokines that are proinflammatory in nature like interleukin 1 and 6, along with tumour necrosis factor (TNF) in the blood, are responsible for increased bone resorption in individuals with diabetes mellitus. Other effects, such as reduced osteoblast proliferation and matrix formation, result in decreased bone production and aggravate bone resorption. Along with all of these variables, there is an increase in osteoclast cell recruitment, which leads to accelerated bone resorption [14-16].

Implant success rates are better in patients with superior bone quality and quantity. The initial BMD not only keeps the fixture mechanically immobilised while healing, but it additionally allows for the distribution of stress and transfer from the prostheses to the bone implant surface [17].

In all areas of the jaws, Misch recognised four categories of BMD (D1 to D4) that differ in both macroscopic trabecular and cortical bone types. The D1 type of bone is dense and homogenous, which has various advantages in implant dentistry. In front regions of mandibular arches with moderate to severe resorption, D1 bone is more common. The D1 bone type has the maximum light microscopic bone contact at the implant interface, at more than 80%. In addition, the apical region of the D1 type of bone generates more heat. In the crestal region of bone, the D2 type of bone is made up of coarse trabecular bone and dense to the porous cortical bone in the interior. The trabeculae of D2 bone are 40%-60% stronger than the trabeculae in the D3 type of bone. In any arch, the D3 bone is most commonly seen in the front maxilla and posterior areas of the mouth. The bone of the D3 type is present in the front region of the maxillary arches and is typically narrower than its D3 mandibular counterpart. D3 bone is not only 50% weaker than D2 bone, but it also has less favourable bone-implant contact. The bone of the D4 type has a low density and little or no mineral content. It's the polar opposite of D1 (dense cortical bone). The posterior part of the maxilla is the most prevalent place for this kind of bone. The trabeculae of D4 bone can be up to ten folds weaker than the cortex [8,18].

Adell et al. [19] in their study stated a 10% higher success rate in the anterior mandible than in the anterior maxilla. Dekker et al. [20] in their study concluded the front regions of edentulous mandibular arches have more and greater quality of bone which is trabecular in nature, which might explain why dental implants in this site have superior primary stability. In the soft bone type, Engquist et al. [21] observed a 78% implant failure. The main stability of the implant, as well as the implant stability quotient, is directly influenced by BMD. Turkyilmaz [17] also concluded that implants can be placed in the D3 type of bone with a significantly high success rate.

Diabetes mellitus is represented by a higher glucose level in the blood. The amount of glycosylated haemoglobin is used to determine glycemic management (HbA1c). The HbA1c level is a three-average of glucose readings [22]. HbA1c values in unaffected healthy people are 4-6. HbA1c values between 6.1 and 8 are considered well-controlled diabetes, whereas levels of more than 8 are considered poorly controlled diabetes. Maintaining a stable HbA1c level is critical for implant life because glycaemic management is directly linked to the generation of complications in patients with diabetes. Sghaireen et al. [23] suggested that T2DM appears to be a minor risk for the survivability of implants, according to the findings. Peled et al. [11] proposed that T2DM shouldn’t affect the health of the mucosa if the condition is effectively managed, despite the fact that it might cause irritation and slow wound healing was hence concluded.

The importance of a pre-surgical evaluation of bone mineral density, as well as knowledge of other anatomical features, is critical for the effectiveness and prognosis of implant therapy. Sghaireen et al. [24], which was aimed at comparing the diagnostic accuracy of CBCT grayscale values with dual-energy x-ray absorptiometry (DXA) values for the detection of osteoporosis, concluded that CBCT values showed a positive correlation with the DXA scores. Hence, it could be used as a significant tool for BMD evaluation. Due to its advantages, such as less radiation exposure and the possibility of exploring structures and anatomic landmarks that are vulnerable to primary implant stability, cone beam computed tomography was chosen as the radiographic evaluation tool. The second reason leading to the rising popularity of cone beam computed tomography scanning is the growing acceptance of computer-guided surgery, which depends on digital planning based on high-quality CBCT pictures and includes generating a 3D virtual dental patient by superimposing intraoral and extraoral facial scans.

In the current study, BMD was assessed by CBCT in the potential implant sites A, B, C, D, and E, in the edentulous mandible, as described by Misch, in individuals with and without T2DM. The mean BMD in individuals with DM was 446.08 HU in the trabecular region compared to 491.77 HU in normal individuals, 1265.69 HU in the buccal cortical plate for patients with T2DM compared to 1267.45 HU in patients without diabetes mellitus, 1479.01 HU in the lingual cortical plate for individuals without T2DM compared to 1259.8 HU in patients with T2DM. All other researchers agree that type 2 diabetes affects bone mineral density in potential implant locations, which validates the conclusions of this study.

Special considerations for implant placement in diabetics

In diabetics, good preoperative and postoperative glucose management is necessary to achieve better osseointegration. Antibiotics that are used for prophylaxis (Table 4).

**Table 4:** Antibiotics that are used for prophylaxis.

A. Intraoral application	B. Intraoral application: nasal or chronic sinus involvement
Penicillin G, 1–2 million U IV q2h	Amoxicillin/clavulanic acid, 650 mg po q4h
Penicillin V, 2 g po q2h	Ampicillin/sulbactam 2g1g IV/IM q3h
Clindamycin, 300 mg po IV q6h	Clindamycin, 300 mg po IV q6h
-	Cephalexin, 500 mg po q4h
-	Cefazolin, 1 g IV/IM q4h

Prophylactic antibiotics have proven to increase the successful placement of implant fixtures in patients with diabetes, and the addition of 0.12% ChX leads to an improvement in its prognosis. The surface of implant features (implants covered using bioactive material) and longer length and success rates of implant prostheses are improved with wider implants, as proven in literature in diabetes patients. Some researchers have discovered promising outcomes in experiments to increase osseointegration; however, the findings have yet to be confirmed in humans. Systemic injection of aminoguanidine was found to minimise the negative effects of diabetes on osseointegration in a few investigations. In Sharma et al.'s study with a diabetic rat's brain, researchers used “recombinant human fibroblast growth factor 2 (rhFGF2)” encapsulated with a “poly glycosylated poly lactide (PGLA)” membranous structure from the lesion of the calvaria, and histomorphic examination revealed normal bone development [25]. In research on a similar premise, Ding et al. employed recombinant rat insulin-like growth factor (rrIGF1) encapsulation done using PGLA around an implant made up of titanium and placed in the calvarial region of rats with diabetes [26].

The limitations of the study are that the study has been carried out in a population with type 2 diabetes. Further studies are recommended to establish the study protocols in a diabetes mellitus type 1 population. The study has utilised CBCT as the diagnostic measure. Other diagnostic measures are required to be studied along with the CBCT.

## Conclusions

CBCT can be considered as a modality for evaluation of the BMD for implant placement. Bone mineral density doesn’t seem to be affected in the buccal cortical region in controlled T2DM when compared to non-diabetics. Slight variations in the BMD are observed in the lingual cortical plate and trabecular region, which still lie in D1 and D3 types of bone, respectively, which allows for successful implant placement. Certain measures for the placement of implant fixtures, specifically in patients with T2DM, are mentioned in the study. The present study evaluates the bone density in T2DM individuals, and further studies are necessary for patients with T1DM and the one with poorly controlled diabetes. Further studies are required for an evaluation of the success rate of implant placement in T1DM.
